# Enhancing Molecular Testing for Effective Delivery of Actionable Gene Diagnostics

**DOI:** 10.3390/bioengineering9120745

**Published:** 2022-12-01

**Authors:** Árpád Ferenc Kovács, Zaránd Némethi, Tünde Abonyi, György Fekete, Gábor T. Kovács

**Affiliations:** 2nd Department of Paediatrics, Semmelweis University, Üllői út 26, 1085 Budapest, Hungary

**Keywords:** actionable genetic diagnosis, nanopore sequencing, long-read sequencing, complex structural variants, single cells, genetic counselling

## Abstract

There is a deep need to navigate within our genomic data to find, understand and pave the way for disease-specific treatments, as the clinical diagnostic journey provides only limited guidance. The human genome is enclosed in every nucleated cell, and yet at the single-cell resolution many unanswered questions remain, as most of the sequencing techniques use a bulk approach. Therefore, heterogeneity, mosaicism and many complex structural variants remain partially uncovered. As a conceptual approach, nanopore-based sequencing holds the promise of being a single-molecule-based, long-read and high-resolution technique, with the ability of uncovering the nucleic acid sequence and methylation almost in real time. A key limiting factor of current clinical genetics is the deciphering of key disease-causing genomic sequences. As the technological revolution is expanding regarding genetic data, the interpretation of genotype–phenotype correlations should be made with fine caution, as more and more evidence points toward the presence of more than one pathogenic variant acting together as a result of intergenic interplay in the background of a certain phenotype observed in a patient. This is in conjunction with the observation that many inheritable disorders manifest in a phenotypic spectrum, even in an intra-familial way. In the present review, we summarized the relevant data on nanopore sequencing regarding clinical genomics as well as highlighted the importance and content of pre-test and post-test genetic counselling, yielding a complex approach to phenotype-driven molecular diagnosis. This should significantly lower the time-to-right diagnosis as well lower the time required to complete a currently incomplete genotype–phenotype axis, which will boost the chance of establishing a new actionable diagnosis followed by therapeutical approach.

## 1. Introduction

A great proportion of genetic disorders manifest phenotypically by early adulthood, resulting in a cumulative incidence of observed rare diseases between 1.5–6.2% in the general population [1,2]. Due to the wide phenotypic heterogeneity and lack of robust molecular testing strategies, diagnosis is challenging and is often delayed by several years. According to the latest telomere-to-telomere human genome assembly, the size of the human genome is of the order of 3.055 Gbp [3]. Besides the relatively large size of the genome, dysfunctional methylation, histone modifications and RNA expression may also elicit a phenotypic burden; therefore, a systematic approach is needed to correctly address genotype–phenotype correlations [4]. Currently, diagnostically used cytogenetics and molecular biology techniques depict only certain types of human genetic alterations (e.g., due to DNA fragmentation during library preparation for next-generation sequencing (NGS), the detection of short tandem repeats, as trinucleotide repeat expansions are not feasible, and also the detection of structural variants is quite limited [5]. The resolution of G-band karyotyping is mostly limited to 3 Mbp, and the higher resolution providing an array-comparative genome hybridization (array-CGH) approach cannot detect low-level mosaicism, balanced translocations or uncover copy number variations where oligoprobes have not been designed in the array) [6]. A comprehensive meta-analysis has shown that the diagnostic utility of the array-CGH and NGS approaches (whole-exome sequencing, whole-genome sequencing) varies between 10–41%, but many cases it still remains unresolved [7]. Therefore, a new comprehensive cytogenomic approach is needed to improve the yield of molecular cytogenomic diagnostics. Long-read nanopore sequencing permits the detection of both nucleotide-level and structural-level variations and methylation pattern alterations, and it is the only method that allows direct RNA sequencing [8].

Here, we reviewed the role of nanopore sequencing within the current molecular testing strategies, its relevance to actionable genetic diagnosis and its impact on genetic counselling. The human genome has a highly complex structure and functions as the backbone of the cell by coordinating the fate of many cellular functions. If critical damage occurs at genomic level, critical cellular functions may occur, mostly in a cell-type-dependent manner, revealing itself in various phenotypic alterations: (1) Some inheritable disorders reveal themselves by the accumulation of a plethora of structural minor anomalies and organ dysfunctions, even at the prenatal, perinatal or early childhood period [9]. A clinical diagnosis and a targeted molecular testing approach should be performed. (2) Most inheritable disorders mask themselves by a subtle phenotype alteration by early childhood, showing unspecific signs and symptoms or age-dependent phenotype penetrance [10]. There is a high need for an effective and time-efficient diagnostic strategy for this group of patients. (3) In the third case, there are patients with underlying genetic disorders with age-dependent/incomplete phenotype penetrance, with mostly the first symptom being the sign of organ dysfunction/a detrimental clinical event. The diagnostic approach and journey for these patients also raises many unanswered ethical questions. The importance of phenotyping accompanied by a detailed genetic anamnesis and at least a four-generation extended pedigree should be utterly prioritized before targeted or high-throughput molecular testing, otherwise the clinical utility of these tests is limited [6,11,12].

## 2. Molecular Testing—Timing and Approach

The timing of molecular testing is of essential importance. The molecular testing of germ line cells is limited. In males, germline cells can be tested from a testis biopsy for (1) nucleotide-level variations in a targeted fashion with Sanger sequencing (SS)/NGS or a nanopore-based sequencing (ONT) approach, or (2) structural-level variations in a targeted fashion with fluorescence in situ hybridization (FISH) with ONT. For a whole-exome or whole-genome approach to detect nucleotide variations, NGS or ONT can be applied. To detect structural variations array-comparative genome hybridization (arrayCGH), ONT or optical genome mapping (OGM) can be applied [13]. For methylation-based analysis, a methylation-sensitive multiple-ligation-based assay (MS-MLPA), pyrosequencing or ONT can be applied. Therefore, ONT provides a comprehensive genome-testing method whilst preserving native nucleic acid modifications [14]. As for preconceptual testing, carrier screening, for most diseases, showing an autosomal recessive inheritance pattern and is available in many countries at a reasonable price. Preimplantation testing during in vitro fertilization and the application of preimplantation gene testing via the detection of pathogenic structural variants in embryos could be of key importance [15]. For targeted prenatal testing, ONT has been successfully applied for the testing of fetal DNA from maternal blood samples [16]. As for postnatal testing, for whole-genome sequencing and diagnosis, a world-record time of 7 h and 18 min has been achieved by the ONT approach [17].

High-throughput deep-sequencing enables the uncovering of variant frequencies and methylation CpG patterns in heterogenous samples. The recently developed nanopore Cas9-targeted sequencing (nCATS) method has proven to provide the aforementioned quality at a targeted level in a cost-effective way [14].

**Figure 1:**
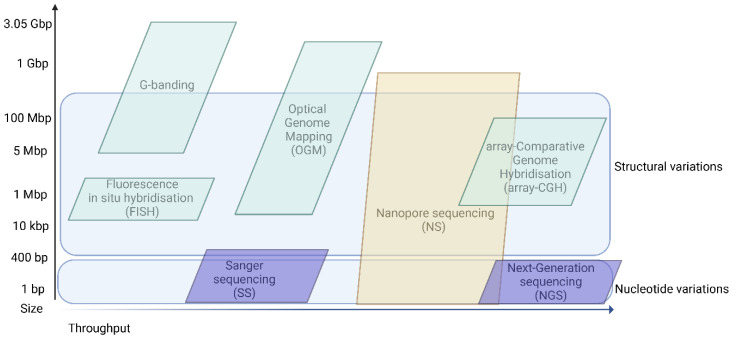
Reliable detection of genetic variants by size and technique throughput. Different techniques allow the detection of certain nucleotides or structural variants. The throughput of the different techniques also varies. Of note, throughput is also instrument-dependent, and this is not reflected entirely in the figure, as in some cases the instrumentation may change the throughput order represented in the figure. Created with BioRender.com (accessed on 6 September 2022).

From a methodological approach, the following two major points need to be addressed to deliver an effective molecular testing strategy: (1) what type of molecular alteration should be detected, and (2) what are the functional consequences of the detected variants? At the DNA level, molecular alterations could be categorized by nucleotide variants and structural variants. Nucleotide variants are usually DNA sequence variations ranging in size between 1–100 bp that may be of critical etiological value in several inheritable disorders and somatic pathogenic variants. Structural variants, ranging in size from several hundred base pairs up to few Mbp, may also elicit inheritable disorders and tumor predisposition or may be of a benign polymorphism value (Figure 1).

## 3. Nanopore Sequencing

**Figure 2:**
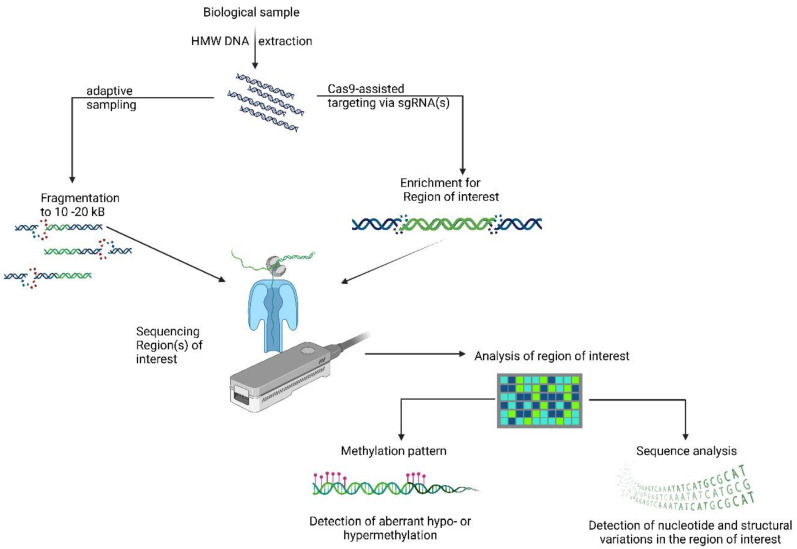
Principles of targeted nanopore sequencing: adaptive sampling and Cas9-assisted methods. High-molecular-weight DNA (HMW DNA) is extracted from relevant biological samples. After quality checking for concentration and purity, two highly potent methods can be applied for a targeted sequencing approach. The adaptive sampling approach can be used for selective enrichment of regions of interest to be sequenced. To enrich prior sequencing during library preparation with designed sgRNAs, the region(s) of interest can be selectively enriched and loaded to the nanopore-based sequencing platform. After quality control assessment, two pipelines can be run, one for methylation pattern analysis, and another for detection of nucleotide and structural variations. This highlights the unique power of nanopore sequencing: parallel detection of both sequence and methylation pattern. Created with BioRender.com (accessed on 6 September 2022).

Recent advances have led to the rapid and highly efficient deciphering of pathogenic variants using nanopore-based sequencing, allowing rapid clinical diagnosis [18]. ONT allows the uncovering of targeted nucleic acid sequences or whole-genome, epigenome (5-methylcytosine), transcriptome and epitranscriptome (N^6^-methyladenine) analysis [18]. Nanoscale-sized nanopores act as biosensors for detecting ionic current changes in real time during single-stranded DNA or RNA molecules (unwound by a motor protein possessing helicase activity) passing in a step-by-step manner [18]. A typical workflow starts with high-molecular-weight DNA extraction coupled with optional fragmentation or size selection (to remove overrepresented small DNA fragments). For library preparation, a relatively short DNA repair and adapter ligation strategy could be used, followed by a loading step on the nanopore-flow cells and real-time sequencing. By applying hybrid error correction tools, the long-read error rate is nowadays between 1-4% of that of short reads. Four main branches could use the advantages of ONT in clinical settings: (1) to identify the background of genetic diseases, (2) to molecularly diagnose cancer patients (e.g., acute leukaemias, solid tumors where certain molecular alterations may greatly influence the therapy of choice [19,20]), (3) rapid pathogen identification in an infectious disease scenario, and (4) to rapidly sequence the major histocompatibility of genes for recipient–donor tissues in transplantation medicine. The strength of nanopore sequencing relies in resolving long-range information, which is one of the main limitations of short-read sequencing technologies [21]. By coupling the unique molecular identifiers (UMI) used in single-cell transcriptomics and genomic long-read transcriptomes, transcriptomes may be sequenced at single-cell resolution with ONT [22]. In Figure 2 we present two methodological approaches for targeted nanopore-based sequencing.

## 4. Where to Fit Long-Read Sequencing in Clinical Genomics?

The relevant milestones that should shape the fitting of long-read sequencing are:

(1) The phenotype-driven molecular testing of both nucleotide variant (e.g., single nucleotide variants, SNV [23,24], short insertion–deletions, indels [25], short tandem repeats (STR) [26], trinucleotide-repeat expansions, etc.) and structural variant detection (e.g., deletions, duplications, cryptic microdeletions/duplications, both balanced and unbalanced translocations [27], gene fusions, complex rearrangements [28], marker chromosomes [29], etc.). Furthermore, methylation pattern detection allows the diagnosis of imprinting disorders as well as haplotyping/phasing [30,31,32] being available when relevant.

(2) Targeted molecular testing in a time-dependent manner for the establishment of an actionable diagnosis, e.g., gene therapy, a modified therapeutical approach and enzyme replacement therapy. Recently, a feasibility study designed to address targeted gene analysis from noninvasive prenatal testing (NIPT) samples using ONT has been described [16]. ONT provides a direct construction of haplotypes, through relative haplotype dosage analysis from maternal blood plasma, which is of importance in diseases such as ß-thalassaemia, spinal muscular atrophy, Duchenne’s and Becker’s muscular dystrophies and Hunter’s disease [16]. As is summarized in Table 1, the molecular diagnosis of actionable rare diseases and haematological malignancies is feasible in a rapid and reliable fashion with ONT. Identified pathogenic variants in the *GLA* (leading to Fabry’s disease), *GBA* (leading to Gaucher’s disease) or *PAH* (leading to phenolketonuria) genes involve a close follow-up, and the affected patients may be eligible for specific enzyme replacement therapy and/ or substrate-reduction therapy [8,33,34]. As for rare disease diagnostics, one of the most common causes of the intellectual disability, Fragile X syndrome, causing STR alterations in the *FMR1* gene as well other genes of interest causing intellectual disability, skeletal or inborn heart disorders, has been successfully detected by the ONT platform. One of the key questions in tumor diagnostics is to identify the actionable gene alterations that may modify the therapeutic strategy in a rapid fashion as well to conclude whether the detected alteration is limited to the somatic tumor-associated tissue or is a germline alteration. The second part of Table 1 depicts such gene alterations that are actionable findings in tumoral settings.

(3) Multiomical and high-resolution-based testing for the depiction of heterogeneity and mosaicism. In several in vitro studies involving cell lines and primary cells, ONT has been successfully applied, providing further evidence of nanopore application for variant analysis (Supplementary Table S1).

The current limitations of ONT include the slightly different approach to DNA extraction and library preparation. The input and quality of the sample DNA may limit the sequencing throughput, and as for post-sequencing, the sequence and methylation pattern need different analysis pipelines. Also the calling of SNVs although has been significantly improved (varying between 1–4%) in recent years, it needs further enhancement to be below 1% of the error rate [5].

**Figure 3:**
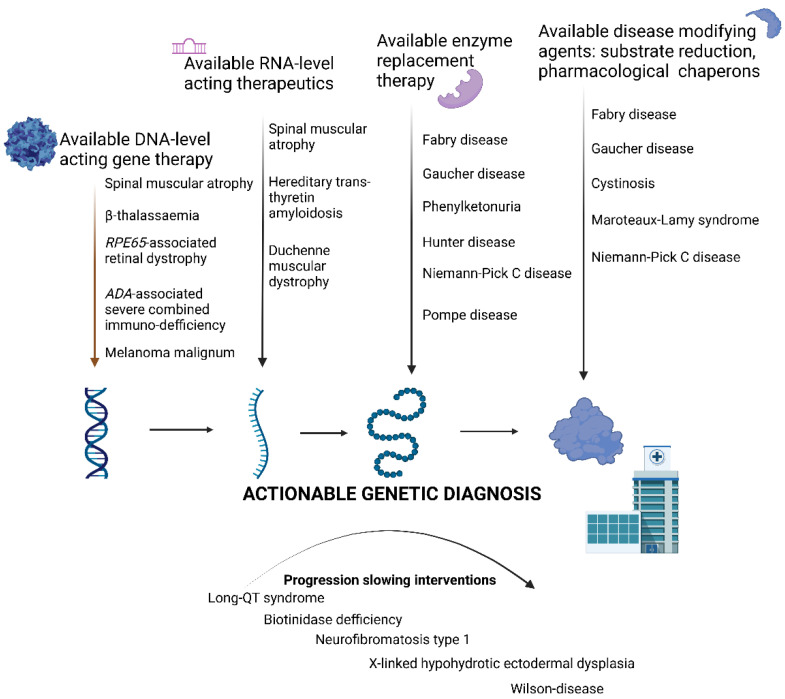
Actionable genetic diagnosis—identification of certain genetic diagnoses opens the pathway for either disease-specific therapy or medical actions that may significantly lower the burden of phenotype and enhance the quality of patients’ life. Created with BioRender.com (accessed on 16 November 2022).

Actionable genetic diagnosis:

The definition of an actionable genetic diagnosis in conjunction with the ACMG’s newest guidelines can be defined as an available medical intervention for certain genetic disorders by reducing morbidity and mortality and enhancing the quality of life [47]. Possible medical interventions arise from a gene-therapeutic point, addressing pathogenic variants at the DNA level in a tissue-specific/tropic fashion. Next, acting at the RNA level, small interfering RNAs or anti-sense oligonucleotides may act at the cellular level to reduce the phenotypic burden. At the protein level, enzyme-replacement therapy is available for quite a few inborn error-of-metabolism disorders, and molecular chaperones enhancing enzymatic activity or substrate-reducing agents with disease-modifying effect are available for selected lysosomal storage disorders. Medical interventions of actionable genetic diagnoses are applied nowadays in the postnatal lifecycle; however, the implementation of disease-modifying therapies in the prenatal period [48] or as a new core concept a possible new era of in utero gene therapy may also arise [49] (Figure 3).

## 5. Genetic Counselling—State of the Art

Genetic counselling reflects the backbone of human genomics analysis by finding the answer to the following three fundamental questions: (1) Why is it recommended to opt for a genetic test? (2) What is our target nucleic acid sequence that should be analyzed by a suitable test available at that particular timepoint, highlighting the strengths and limitations of it? (3) How should we critically interpret and obtain insight about the data provided by the genetic analysis?

One of the most challenging tasks of genetic counselling is to maximize the clinical utility and, at the same time, minimize the uncertainty of information [50]. This could be enhanced by setting up multidisciplinary professional healthcare teams who can synthesize and collaborate to precisely define and follow up phenotypic spectra, which may maximize the uncovering of phenotype–genotype associations. Thus, the clinical pathway of patients can be significantly improved, often influencing the screening strategies and/or therapeutic approach.

## 6. Role of Pre-Test Genetic Counselling

To define the genotype–phenotype correlation as precisely as possible, a detailed phenotyping and pedigree building is essential, which can also be enhanced by artificial intelligence (AI). Phenotyping begins with a detailed genetic anamnesis. This should include a preconceptual anamnesis (age of the biological mother and biological father at conception, mode of conception and use of periconceptual vitamins) and a prenatal anamnesis (use of or contact with teratologic agents during the embryonal and/or fetal critical period. TORCH-screening, pathological ultrasound findings, the use of prenatal vitamins, drug use, and prenatal genetic tests such as NIPT, G-banding, chromosomal microarray or whole-exome sequencing could be conducted) [51]. The anamnesis of the perinatal period should cover the mode of delivery, possible hypoxic events, the age of gestation, the weight, height and head circumference at birth, the APGAR score, neonatal cardiorespiratory adaptation, the use of O_2_ therapy, newborn hearing screening, newborn feeding difficulties and breastfeeding. With a special emphasis on the advances of developmental milestones and the use of early childhood development therapies, language, social skills and learning ability development should be questioned. At the end of genetic anamnesis, the building of a four- or five-generation genetic pedigree is advised. For the digitalization of pedigrees, different software packages (e.g., Evagene Clinical—a free open-source software available at www.evagene.com (accessed on 6 September 2022) or GenoPro Waterloo, Ontario, Canada—a paid software option available at www.genopro.com (accessed on 6 September 2022)) can be used. Critical questions should be covered during the pedigree assessment such as (1) the spontaneous abortion history of the index patient’s mother and maternal grandmother; (2) any known perinatal death in the family; (3) the occurrence of sudden cardiac death in the family; (4) any malignant tumor development before the age of 45 in the family; (5) any recurring deep-vein thrombosis, any pulmonary emboly in the family; (6) any consanguineous marriage in the family; and (7) any infertility in the family.

**Figure 4:**
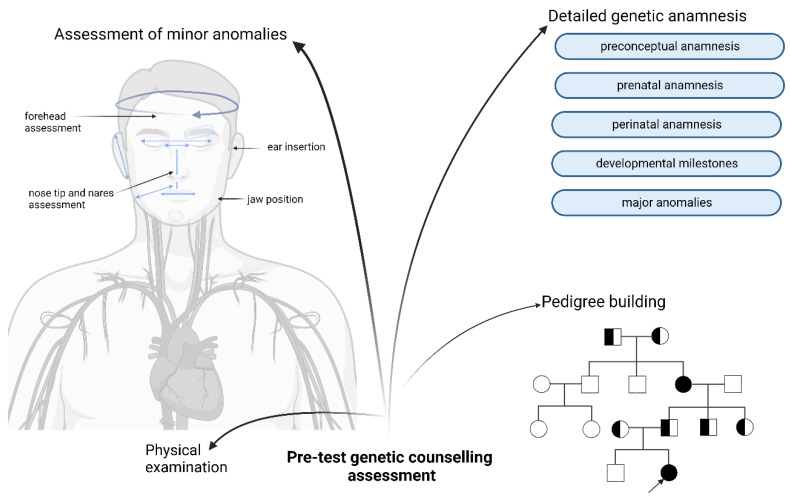
Role of modern genetic counselling—during pre-test counselling, the goal is to elucidate as far as possible the phenotype alterations and to assess the family tree in order to choose the adequate genetic tier test. Created with BioRender.com (accessed on 8 September 2022).

Next, a genetic physical assessment should include the detailed phenotyping of minor anomalies. Detailed phenotyping is an important way to evaluate the impact of penetrance, the possibility of uncovering a second genetic alteration and of expanding the phenotypic variability of a molecular finding [52,53,54,55]. The use of prenatal data may also enhance the findings of a high-throughput molecular analysis [51]. If a targeted approach is used, the detailed phenotype will determine the test of choice [56]. Guidelines on the standard use of systematic phenotyping are available [57,58,59]. The use of artificial intelligence for the refinement of minor anomalies (e.g., Face2Gene—a free open-source online platform [60]) can also be useful. Systematic phenotyping includes the depiction of facial minor anomalies, which should include the size of the nasal bridge, a nasal tip and nares assessment, canthal folds, endocanthal lengths, interpupillary distance, exocanthal length, palpebral fissure slanting, eyelash length and density, eyebrow thickness and/or conjoined eyebrows, philtrum size, lower and upper lip thickness and slanting, intercommissural distance, enlarged interdental space, a high narrow palate, bifurcated uvula, tongue size and asymmetry, bilateral philtrum–mandibular angle distance, forehead size and protrusion, bitemporal distance, ear set height, ear asymmetry, rotation of the external ear, jaw position and mandibular size. Next, the shape and size of the skull as well as the hairline insertion and hair thickness anomalies should be assessed. In addition, skin alterations (e.g., café-au-lait spots, angiokeratomas, etc.) and hand and feet minor anomalies should be assessed in addition to a regular physical examination. A detailed depiction of minor anomalies and their annotation should be performed according to the human phenotype ontology (HPO) [61]. Next, the objectifying of minor anomalies by precise measurements during minor anomaly depiction should be calculated for age- and gender-specific percentile values. In addition, a detailed depiction of growth curve tendencies by a comparison of percentiles specific to age, gender and diagnosis (e.g., growth percentiles for girls between age 0–2 diagnosed with Down’s syndrome) should be conducted. Based on the evaluation of the detailed genetic anamnesis, physical examination and pedigree, the best-suited targeted or high-throughput analysis should be chosen. Detailed information about the usefulness, benefits and limitations of the proposed test should be provided. Then, informed consent should be signed, with special emphasis on the reporting of secondary findings according to the latest ACMG guidelines [47] (Figure 4).

## 7. Role of Post-Test Genetic Counselling

The role of post-test genetic counselling is a critical evaluation of the results of the applied genetic test in light of the phenotype. The evaluation of genotype alterations should be in line with the observed phenotype. It is recommendable, when possible, that the positive findings be confirmed by another technique. Of note, if variants of unknown significance have been identified or the methylation pattern has also been determined in addition to sequencing, it could be re-evaluated in the near future as more data on variants of unknown significance start to shed light on either their benign or pathogenic effect. As the genotype–phenotype correlation that has been established the genetic medical report should contain the precise genetic alteration both at the cytogenetic and molecular nomenclature (preferably according to the latest assembly, e.g., GRCh38), the accession number/link of the detected variant, the interpretation of the variant at the cellular level and the next detailed clinical significance should be provided. In the detailed clinical significance, the possible therapeutical approaches and follow-up strategy should be written and discussed with the patient and/or legal guardian by taking into account the national human genetic laws of a specific country.

## 8. Concluding Remarks

The precise uncovering of our genetic information of interest should be one of our highest priorities, which should be conducted by providing the most complete molecular landscape in order to establish next-generation genetic counselling and guidance. As technology advances, AI has become more prevalent in molecular medicine, and genetic data has become more prone to being sequenced at larger and larger scales. Timing is of the essence to molecular diagnosis, as the patient will have the highest opportunity to benefit from specific medical care and/or treatment the earlier the diagnosis is conducted. Precise and detailed phenotyping should be performed in order to conclude the correct genotype–phenotype correlation and medical care to be provided as soon as possible for every actionable genetic diagnosis.

## Figures and Tables

**Table 1:** Clinically actionable gene alterations successfully detected by ONT from processed human samples.

Region of Interest	Sample	DNA/RNA Extraction	Library Prep	Sequencing Platform	Results	Validation	Reference
Genetic regions of interest for inborn errors of metabolism							
*GLA*	Blood	Flexigene DNA kit (Qiagen)	PBK004	MinION	Detected *GLA* nucleotide variations	Known *GLA* variants were sequenced	[33]
*GBA*	Blood	Phenol-chloroform salting out	LSK-109	GridION	Detected variants, SNV	SS	[34]
*PAH*	Blood, saliva, fibroblasts	NA	LSK-109	GridION	Successful SV, complex rearrangements, SNV detection with adaptive sampling	SS, Southern blot	[8]
Genetic regions of interest for intellectual disability							
*TAF1*	Blood	DNA Midi kit (Qiagen)	LSK-109	GridION	Detected repeat expansions	Fluorescence-based PCR	[35]
* C9orf72, FMR1 *	hiPSC from patients	Phenol-chloroform extractions	LSK-108 or LSK-109	MinION	STR	Southern blot	[36]
* RFC1, NOTCH2NLC, FXN, AR, DMPK *	Blood	Qiagen Gentra PureGene blood kit (NSW) or QIAsymphony DSP DNA Midi Kit	LSK109 or LSK110	MinION or GridION	STR and methylation profiling	RP-PCR and Southern blot	[37]
Genetic regions of interest for skeletal and or heart disorders							
*DMD*	Blood and saliva	DNA extraction	LSK-109 or LSK-110	MinION or GridION	Detected SVs and SNVs	SS	[38]
*ALMS1, DMD, ABCA4, AGL, XYLT1 and other ROI*	Blood, saliva, fibroblasts	NA	LSK-109	GridION	Successful SV, complex rearrangements, SNV detection with adaptive sampling	SS, Southern blot	[8]
Genetic regions of interest tumour predisposition							
*PML-RARA*	Blood and bone marrow	Promega Maxwell Instrument	LSK-108	MinION	Gene fusion detection	NGS—Illumina	[39]
*IGHV*	Blood	QIAamp DNA blood mini kit	LSK-109	MinION	Detection of IGHV small subclones	SS	[40]
*BRCA1, KRT19, BRAF, KRAS, TP53*	Breast tumor	MasterPure kit (Lucigen)	LSK-109	MinION	SNV, SV, methylation by Cas9-approach	Illumina WGBS	[14]
*HLAB2*	Blood	Guanidium–HCl based chloroform extraction	NSK007	MinION	HLA-B genotyping	SS	[41]
*BCR-ABL1, FGFR2 fusions,*	Hematologic and solid tissue specimens	RNA extraction Promega	LSK-108	MinION, Flongle	Gene fusion detection	NGS	[42]
* EGFR *	Blood plasma	QIAamp Circulating Nucleic Acid Kit	LSK-109	MinION	*EGFR* amplifications	NGS, SS	[43]
* NPM1, FLT3, CEBPA, TP53, IDH1 and IDH2 *	Bone marrow	QIAamp DNA Blood mini kit	LSK-108	MinION	SNV, indel detection	SS	[20]
* BCR-ABL1 *	Blood	Blood genomic DNA mini kit	LSK-108	MinION	Specific positions of translocation	SS	[44]
* FLT3 *	Blood	NA	LSK-108	MinION	*FLT3* allelic mutations	NGS—Ion Torrent	[45]
mtDNA							
* mtDNA *	Blood	QIAamp Mini Blood Kit	NSK007 or RAD001	MinION	SNV, homopolymer, dinucleotide repeat	NIST-traceable mtDNA sequencing standard	[46]

NA—no available data on the used kit, NGS—next-generation sequencing, SS—Sanger sequencing, SV—structural variation STR—short tandem repeats.

## Data Availability

Not applicable.

## Supplementary Materials

The following supporting information can be downloaded at: https://www.mdpi.com/article/10.3390/bioengineering9120745/s1, Table S1: Relevant data on human cell line samples processed by ONT. (References [62,63,64,65,66] are cited in the supplementary materials).

## Abbreviations

ACMG	American College of Medical Genetics and Genomics
AI	artificial intelligence
arrayCGH	array comparative genome hybridization
CRISPR	clustered regularly interspaced short palindromic repeats
FISH	fluorescence in situ hybridization
HMW DNA	high-molecular-weight DNA
HPO	human phenome ontology
nCATS	nanopore Cas9-targeted sequencing
MS-MLPA	methylation-sensitive multiple ligation-based assay
NGS	next-generation sequencing
NIPT	noninvasive prenatal testing
OGM	optical genome mapping
ONT	nanopore-based sequencing
SNV	single nucleotide variation
SS	Sanger sequencing
STR	short tandem repeats
TORCH	toxoplasmosis, others, rubella, cytomegalovirus and herpes simplex virus
