# Supplementary material for: Enhancing Molecular Testing for Effective Delivery of Actionable Gene Diagnostics
Source: Bioengineering (Basel). 2022 Dec 1;9(12):745. doi: 10.3390/bioengineering9120745 (PMC9774983; doi:10.3390/bioengineering9120745)
Supplement: Supplementary file 1 [file bioengineering-09-00745-s001.zip › bioengineering-1938721-supplementary.pdf]

bioengineering

MDPI

*Review*

# Enhancing Molecular Testing for Effective Delivery of Actionable Gene Diagnostics

**Árpád Ferenc Kovács *, Zaránd Némethi, Tünde Abonyi, György Fekete and Gábor T Kovács**

2nd Department of Paediatrics, Semmelweis University, Üllői út 26, Budapest 1085, Hungary
* Correspondence: kovacs.arpad@med.semmelweis-univ.hu

**Table S1.** Relevant data on human cell line samples processed by ONT.

| Region of interest | Sample | DNA extraction | Library Prep | Sequencing platform | Results | Validation | Reference |
|---|---|---|---|---|---|---|---|
| *ABCB1* | THP-1 cell line | QIAamp DNA mini kit | LSK108 or LSK 109 | MinION | Structural variants, *ABCB1* translocation detected by nanopore sequencing | Chip seq, NGS | [62] |
| Chromosome 1 | Lymphoblastoid cell line | Chromosome preparation | LSK-109 | MinION | Highly continuous and selective assembly of chromosome 1 | NA | [63] |
| *Gene fusion BCR-ABL1 PAX5-AUTS2 HAS2-PLAG1* | K562 cell line | RNA - RNeasy system | LSK-108 | MinION | Fusions were detected | NGS - Illumina | [39] |
| Short tandem repeats | HiPSC line | HMW DNA preparation with a modified phenol-chlorophorm method | LSK-108, 109 | MinION, PromethION | STR expansions | Southern-blot | [36] |
| NMD-targeted isoforms | HeLa cell line | Total RNA: GenElute Mammalian Total RNA Miniprep Kit | DCS-108 | GridION | identification of many novel NMD-sensitive mRNAs | NGS -Illumina | [64] |
| Evaluate CpG methylation | GM12878 lymphoblast cell line | Phenol-chlorophorm | LSK-108 | MinION | NS allows to generate human epigenome | Bisulfite sequencing | [65] |
| *TP53, BRAF, KRAS* | MDA-MB-231 , MCF-7, MCF-10A, GM12878 | Circulomics CBB kit | LSK-109 | MinION | SNV, methylation | whole-genome bisulfite sequencing | [14] |
| *whole genome* | GM12878 | Qiamp DNA Mini kit (Qiagen) | LSK-108, RAD002 | MinION | assembly of human genome 99,88% | Non-validated | [66] |

NA—no available data, NGS—next generarion sequencing.

## References

62. Williams, M.S.; Basma, N.J.; Amaral, F.M.R.; Williams, G.; Weightman, J.P.; Breitwieser, W.; Nelson, L.; Taylor, S.S.; Wiseman, D.H.; Somervaille, T.C.P. Targeted nanopore sequencing for the identification of ABCB1 promoter translocations in cancer. *BMC Cancer* **2020**, *20*, 1075. https://doi.org/10.1186/s12885-020-07571-0.
63. Kuderna, L.F.K.; Solís-Moruno, M.; Batlle-Masó, L.; Julià, E.; Lizano, E.; Anglada, R.; Ramírez, E.; Bote, A.; Tormo, M.; Marquès-Bonet, T.; et al. Flow Sorting Enrichment and Nanopore Sequencing of Chromosome 1 From a Chinese Individual. *Front. Genet.* **2020**, *10*, 1315. https://doi.org/10.3389/fgene.2019.01315.
64. Karousis, E.D.; Gypas, F.; Zavolan, M.; Mühlemann, O. Nanopore sequencing reveals endogenous NMD-targeted isoforms in human cells. *Genome Biol*. **2021**, *22*, 223.
65. Lee, I.; Razaghi, R.; Gilpatrick, T.; Molnar, M.; Gershman, A.; Sadowski, N.; Sedlazeck, F.J.; Hansen, K.D.; Simpson, J.T.; Timp, W. Simultaneous profiling of chromatin accessibility and methylation on human cell lines with nanopore sequencing. *Nat. Methods* **2020**, *17*, 1191–1199. https://doi.org/10.1038/s41592-020-01000-7.
66. Jain, M.; Koren, S.; Miga, K.H.; Quick, J.; Rand, A.C.; Sasani, T.A.; Tyson, J.R.; Beggs, A.D.; Dilthey, A.T.; Fiddes, I.T.; et al. Nanopore sequencing and assembly of a human genome with ultra-long reads. *Nat. Biotechnol.* **2018**, *36*, 338–345. https://doi.org/10.1038/nbt.4060.
